# Assessing Heat-Related Mortality Risks among Rural Populations: A Systematic Review and Meta-Analysis of Epidemiological Evidence

**DOI:** 10.3390/ijerph15081597

**Published:** 2018-07-27

**Authors:** Emmanuel A. Odame, Ying Li, Shimin Zheng, Ambarish Vaidyanathan, Ken Silver

**Affiliations:** 1 Department of Environmental Health, East Tennessee State University, Johnson City, TN 37614, USA; odamee@etsu.edu (E.A.O.); liy005@mail.etsu.edu (Y.L.); 2 Department of Biostatistics and Epidemiology, East Tennessee State University, Johnson City, TN 37614, USA; zhengs@mail.etsu.edu; 3 National Center for Environmental Health, Centers for Disease Control and Prevention, Atlanta, GA 30341, USA; rishv@cdc.gov

**Keywords:** rural, mortality, heat-related, vulnerability, systematic review, meta-analysis

## Abstract

Most epidemiological studies of high temperature effects on mortality have focused on urban settings, while heat-related health risks in rural areas remain underexplored. To date there has been no meta-analysis of epidemiologic literature concerning heat-related mortality in rural settings. This study aims to systematically review the current literature for assessing heat-related mortality risk among rural populations. We conducted a comprehensive literature search using PubMed, Web of Science, and Google Scholar to identify articles published up to April 2018. Key selection criteria included study location, health endpoints, and study design. Fourteen studies conducted in rural areas in seven countries on four continents met the selection criteria, and eleven were included in the meta-analysis. Using the random effects model, the pooled estimates of relative risks (RRs) for all-cause and cardiovascular mortality were 1.030 (95% CI: 1.013, 1.048) and 1.111 (95% CI: 1.045, 1.181) per 1 °C increase in daily mean temperature, respectively. We found excess risks in rural settings not to be smaller than risks in urban settings. Our results suggest that rural populations, like urban populations, are also vulnerable to heat-related mortality. Further evaluation of heat-related mortality among rural populations is warranted to develop public health interventions in rural communities.

## 1. Introduction

Most epidemiological studies of the negative impacts of high temperature on human health have focused on urban settings [1,2,3,4,5,6]. However, heat-related health risks among rural populations remain underexplored. The adverse health impacts of high temperatures on urban populations have been attributed to several factors [7,8,9]. Of those, the urban heat island (UHI) effect, which can be defined as a phenomenon where surface temperatures in urban areas are higher than surrounding rural areas [10,11,12], and heterogeneity in socioeconomic characteristics are noteworthy [9,13].

Despite the fact that rural locations are often cooler than urban centers, rural areas may be distinctly disadvantaged in factors that increase population vulnerability to extreme weather, such as social isolation, access to health care and air conditioning, and baseline health status, with some factors being markedly worse in less developed regions. To be able to formulate comprehensive heat-health action plans, it is imperative that we assess heat-related health risks in rural areas [14]; however, conducting risk assessments for rural settings can be challenging. Most rural areas, especially in underdeveloped countries, lack meteorological data due to a paucity of weather monitoring stations [15,16]. Additionally, relatively small rural populations militate against epidemiologic studies with sufficient statistical power.

Moreover, the definition of rural areas remains vague and existing definitions depend on that of urban areas [17]. There is no single, universally preferred definition of rural, nor is there a single rural definition that can serve all policy purposes [18]. Thus, a rural area defined in a developed nation may differ from another in a less developed country in terms of metrics of population density, infrastructure, and resources.

Even though fewer studies have examined the temperature-mortality relationship in rural areas, some studies in this category have reported that people in less urban areas may be more susceptible to heat [16,19,20,21]. There is also emerging evidence regarding high rates of heat-related illness in rural areas [22]. Overall, vulnerability to climate change is a function of exposure, sensitivity and adaptive capacity [23], making isolated rural populations with inadequate infrastructure more vulnerable to heat-related mortality. However, no study to date has systematically assessed the current global epidemiologic evidence related to rural vulnerability to summer heat in the peer-reviewed heat-related mortality literature.

The goal of this study is thus to conduct a systematic review of the epidemiologic literature of the association between high temperature and mortality in rural populations and generate the synthesis of results from different studies across the globe, using meta-analysis to examine rural vulnerability to heat-related mortality worldwide.

## 2. Materials and Methods

### 2.1. Search Strategy and Screening Criteria

We conducted a systematic literature review in April 2016 and revisited the literature in May 2017, and again in April 2018 to update our search. We used scientific peer-reviewed search engines PubMed, Web of Science, and Google Scholar with no restriction on the geographical location or period of publication. Keywords used for this review were: (Rural OR “non-urban”) AND (high temperature OR heat OR hot weather OR climate) AND (mortality OR deaths) AND (relative risk OR risk ratio OR effect measure OR change OR “RR”). There were no restrictions on publication date or location of studies.

We manually screened the abstracts of all studies initially located through the search and excluded the following:

1. Studies not published in English;
2. Studies not performed on human populations (non-epidemiological studies);
3. Studies reporting no effect estimates (i.e., relative risks (RRs) or % change in mortality), those reporting effect estimates only for subpopulations, such as the elderly, but not for the entire population in the study area;
4. Commentaries, review articles, and editorials;
5. Studies on morbidity; and
6. Studies focusing on extreme temperature (heat waves), due to their inconsistent definitions [24,25] and occurrence within short time periods [26].

### 2.2. Data Extraction

The effect estimate (RR or % change in mortality) reported in each study was extracted. When effect estimates for multiple lag periods were reported in a study, we selected the estimate for the shortest lag period, usually 0–1 day, due to the acute nature of high temperature effects [26]. Studies have reported that longer lag periods are likely to result in overestimation of the effects, especially when distributed lag non-linear models (DLNMs) are used [27,28]. Due to differences in temperature metrics, studies that used mean daily temperatures were separated from those that used daily maximum temperatures. We normalized and converted all effect estimates and their 95% confidence intervals (CIs) into relative risks per Celsius degree (RRs per °C) increase in temperature for unification purposes, to be able to combine them into an overall RR estimate. The random effects model, which assumes that different studies were drawn from different populations with unique conditions that could impact on the treatment (i.e., temperature) effect, was preferred to the fixed effects model since each study was conducted in a different rural setting and under different conditions. Moreover, the random-effects model ensures that the different effect sizes in all studies are represented in the summary estimate [29].

We further stratified the selected studies into groups based on their level of development, per the United Nations’ classification system [30]. According to the Development Policy and Analysis Division (DPAD) of the Department of Economic and Social Affairs in the United Nations Secretariat, all countries can be classified into one of these broad categories based on the prevailing economic conditions: Developed economies, economies in transition, and developing economies. We then performed a sensitivity analysis to examine whether the level of development was a factor that affects the association between high temperature and mortality.

Both the Cochran’s Q and the I^2^ statistics can be used to determine statistical heterogeneity in the results. The Cochran’s Q, calculated as the weighted sum of squared differences between individual study effects and the pooled or summarized effect across studies, is the traditional measure of heterogeneity, while the I^2^ statistic explains percentage of variation across studies that is due to heterogeneity rather than chance [31,32]. The relationship can be summarized by the equation: I² = 100% × (Q − df)/Q; where df is the degrees of freedom, defined as the number of studies used minus 1. For this study, we relied on the I^2^ statistic, since it is more interpretable, provides more accurate estimates, and is more independent of the number of studies used in the analysis than the Cochran’s Q [32]. All statistical analysis was performed using Comprehensive Meta-Analysis software (Version 3.0, Biostat, Englewood, NJ, USA).

## 3. Results

**Figure 1:**
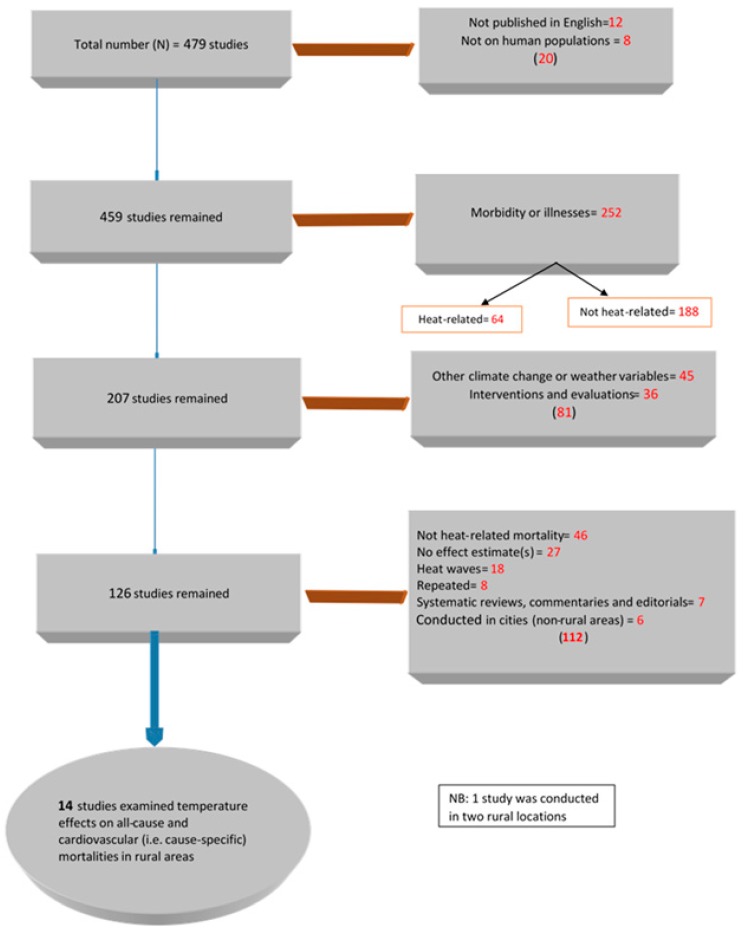
Flow chart illustrating study selection.

The literature search generated 479 studies. Figure 1 presents the selection and exclusion of studies. Among the 459 studies retained based on the first two exclusion criteria, 252 studies were excluded because they studied morbidity instead of mortality. Among the 207 remaining studies, 45 were excluded because they relied on climate variables other than temperature, and 36 were excluded because they focused on interventions and evaluations. The remaining 126 studies were also scrutinized: 46 on other causes of mortality not related to heat, 27 with no effect estimates, 18 on heat waves, and eight duplicated studies were all excluded. Further, seven systematic reviews, commentaries, and editorials and six studies conducted in non-rural settings were also excluded.

**Figure 2:**
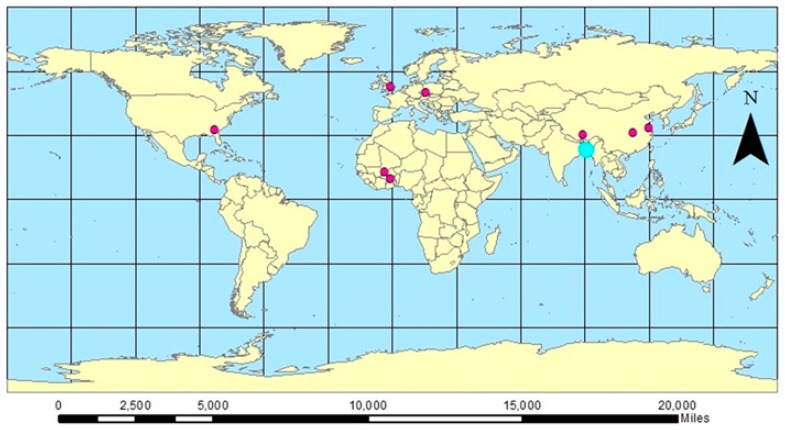
Rural locations covered in this study. * Blue dot represents all 3 studies conducted in Bangladesh (i.e., same location).

As a result, 14 studies that examined the effects of high ambient temperature on mortality were identified. Table 1 presents the characteristics of these 14 studies. All studies were published in the last eleven years between 2006 and 2017. Two studies were conducted in North America (United States), two in Europe (England and Wales, Czech Republic), eight in Asia (China, Bangladesh, India), and two in Africa (Ghana, Burkina Faso). Among the 14 studies identified, eleven used daily mean for temperature metric, two used daily maximum and one used weekly mean (Table 1). In conducting the meta-analysis, we only included the eleven studies that used daily mean temperature since the other two temperature metric groups contain insufficient numbers of studies. The geographical locations of these eleven studies are shown in Figure 2.

### 3.1. Meta-Analysis

**Figure 3:**
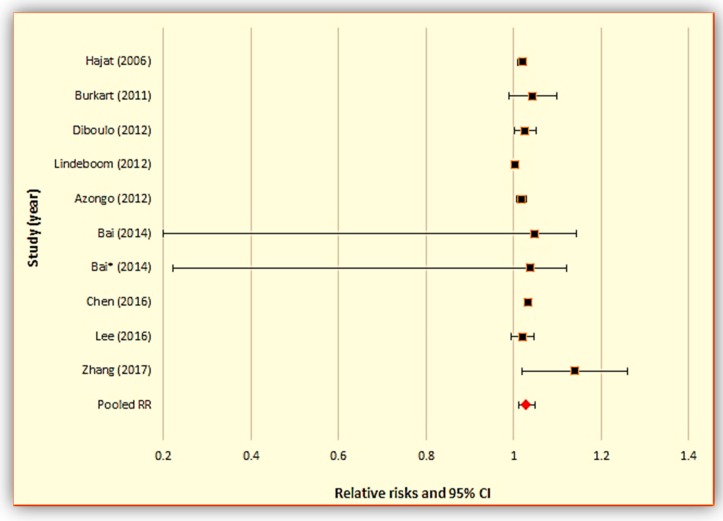
Meta-analysis results for studies using daily mean temperature for all-cause mortality.

**Figure 4:**
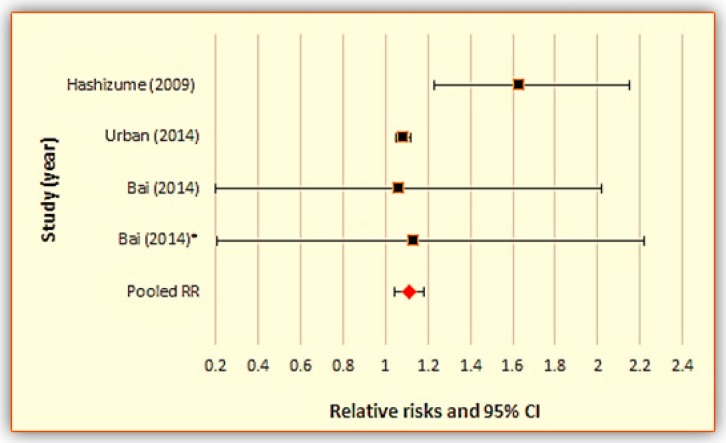
Meta-analysis results for studies using daily mean temperature for cardiovascular mortality.

We conducted two separate meta-analyses for studies examining the relationship between daily mean temperature and (1) all-cause mortality (Figure 3 and Table 2), and (2) cardiovascular mortality (Figure 4 and Table 3), respectively.

The combined relative risks (RRs) for all-cause and cardiovascular mortality were, 1.030 (95% CI: 1.013, 1.048) and 1.111 (1.045, 1.181) per 1 °C increase in mean daily temperature, respectively. This means that in predominantly rural locations, every 1 °C increase in mean daily temperature is associated with 3.0% excess mortality and 11.1% excess cardiovascular mortality. Also, the I^2^ statistics for all-cause and cardiovascular mortality were 0.0% (*p* < 0.05) and 59.4% (*p* < 0.05), respectively, indicating no observed heterogeneity among the all-cause mortality studies and considerable heterogeneity among the cardiovascular mortality studies (I^2^ > 50%).

### 3.2. Sensitivity Analysis

**Figure 5:**
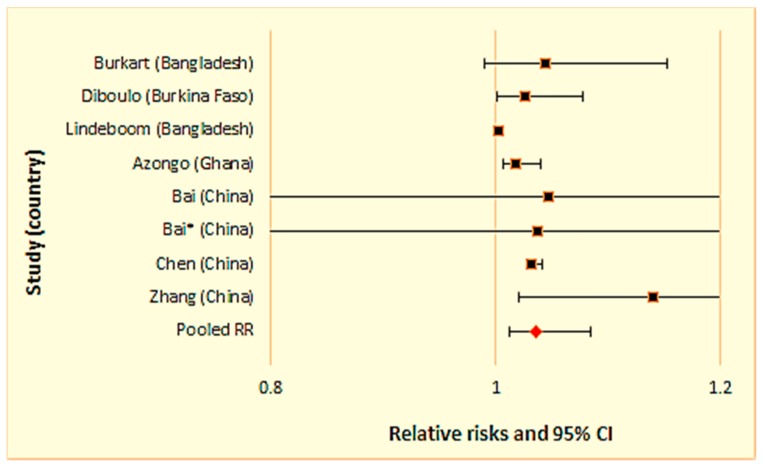
Sensitivity analysis for studies conducted in developing countries.

**Figure 6:**
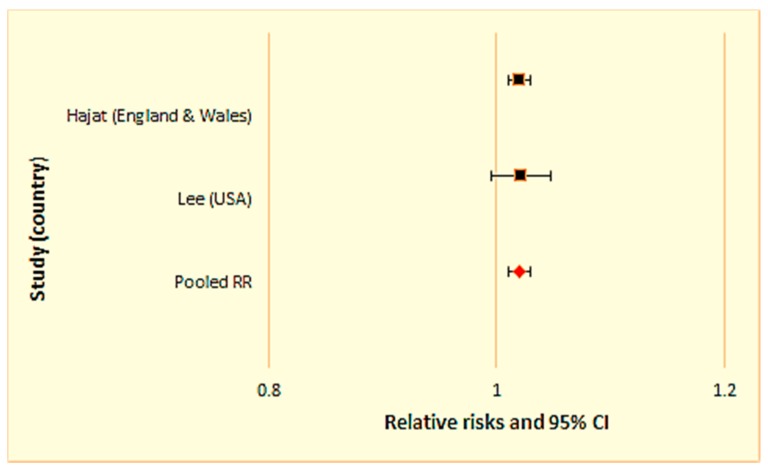
Sensitivity analysis for studies conducted in developed countries.

In addition to grouping studies based on their mortality outcomes (i.e., all-cause and cardiovascular mortality), we also stratified studies into two categories based on the level of economic development of the study nation: Developed and developing countries, using the United Nation’s country classification [31]. None of the selected studies were in countries with transition economies based on the UN classification. The United States and United Kingdom fall into the developed country category and were separated from studies conducted in the remaining countries, all classified as developing countries. A sensitivity analysis was then performed within the developing (Figure 5 and Table 4) and developed country groups (Figure 6 and Table 5). The results show that the excess mortality risk was higher among developing nations (3.6%) than developed nations (2.0%), although there were only two studies in the developed nation group.

## 4. Discussion

Global epidemiological studies of the association between high temperature and mortality have been primarily focused on urban areas, whereas fewer studies to date have examined nonurban areas. To the best of our knowledge, this is the first meta-analysis of heat-related mortality risks in rural locations worldwide. In this study, we focused on the endpoints of all-cause and cardiovascular mortality. We did not include studies of excess mortality during short-term heat waves, typically lasting a few days or weeks, owing to their inconsistent definitions, designs, and methods [24,25]. For the studies included in our meta-analysis, the duration of observations ranged from 4 to 27 years (median = 7 years).

The selected studies used consistent definitions and research designs in which daily mortality was regressed on daily mean temperature. Daily mean temperature is very useful in assessing the temperature-mortality relationship [27,44]. Apart from its ability to provide easily interpretable results, it best represents the temperature exposure throughout the whole day and night [45,46]. A study of ambient temperature and mortality in Wuhan, China suggested that daily mean temperature was the best temperature metric for predicting temperature effects on cause-specific mortality [28]. Further support for use of this metric is that it has the lowest Akaike’s information criterion for quasi-poison (Q-AIC) values, making it a better predictor than the maximum and minimum temperatures [39]. Additionally, it correlates more strongly with mortality than either T_max_ or T_min_ [15] and shows coherent behavior with respect to mortality at both low and high temperatures [37]. Moreover, it is less prone to measurement error [37]. Interestingly, other studies [20,47] reported similar results regardless of the temperature metric used.

In controlling for potential confounding factors, most of the selected studies adjusted for seasonality and time trends. Only a few adjusted for other environmental hazards, such as air pollutants, consistent with the observation of Madrigano [42] that not all studies adjust for ozone as a confounding factor when assessing temperature-mortality relationships. Exposure to ambient air pollutants, mainly particulate matter (PM) and ozone, have been linked to premature mortality [48], and thus may confound the temperature-mortality association [26,49]. Ozone is a summer pollutant and climate change is projected to detrimentally affect ozone air quality and consequently increase mortality [50]. The observed correlations of PM concentrations with temperature are weaker than for ozone [51], yet PM has been found to peak in the summer in certain regions, such as the East Coast of the U.S. [26]. Therefore, PM also may be a confounder for the association between temperature and mortality in these regions. Noting that air pollutants and temperature have different biological effects, Guo [47] posited that their effects are likely to be independent of each other. In summary, the results for confounding or effect modification by air pollutants on the temperature-mortality relationship remain mixed [26].

**Figure A1:**
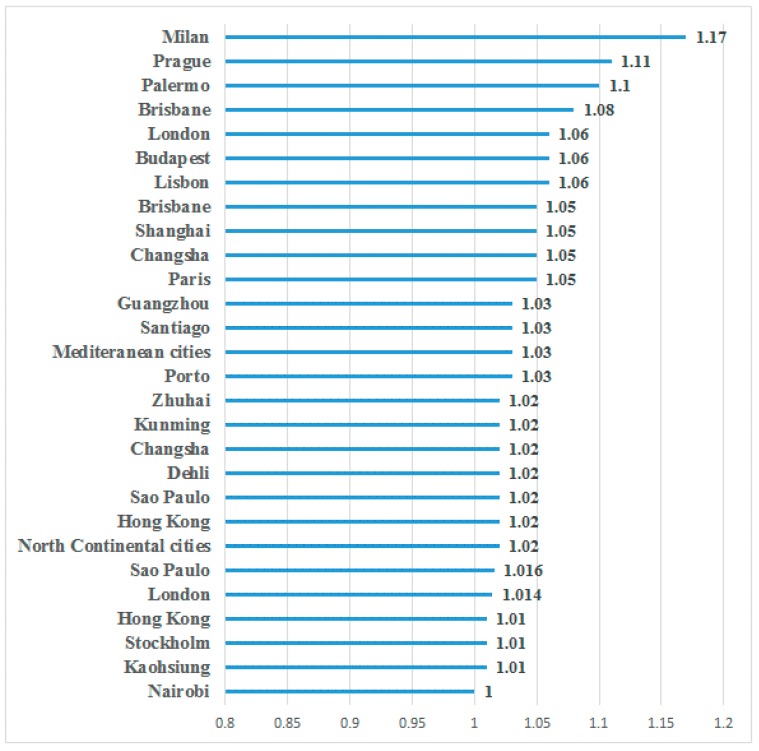
Relative risks (RR) for heat-related all-cause mortality studies conducted in large urban areas (Unit: RR per 1 °C increase; Data adopted from Benmarhnia et al., 2015 [24]).

Our results indicate evidence of heat vulnerability in rural areas. We estimated a 3% excess in all-cause mortality and an 11% excess in cardiovascular mortality to be associated with a 1 °C increase in mean ambient temperature. In comparing the excess heat-related mortality risk with studies in the world’s urban centers, we used the relative risks from a review [24] and illustrated the RRs in Figure A1 in the Appendix. As shown in Figure A1, all-cause mortality RRs per 1 °C increase in large urban areas ranged from 1.00 to 1.17, with more than half of the estimates falling into the range of 1.01–1.03. Therefore, the excess risks in rural areas are similar to those in urban areas. Our results suggest that rural residents may not be less vulnerable to heat. Rural populations may benefit from the absence of extreme heat or “heat island” in rural environments, attributable to larger numbers of water bodies, trees, greenery fields, and lower population density [36]. On the other hand, heat vulnerability is not only a product of heat exposure factors but, more importantly, sensitivity and adaptive capacity [52]. Among rural inhabitants, marked differences exist in the components of heat vulnerability, such as lack of health care infrastructure and access to air conditioning, social isolation, informal settlements, and worse baseline health status. Moreover, certain occupational groups residing in rural areas, such as agricultural workers who spend a great deal of time exposed to extreme temperatures, may be a factor that contributes to increased susceptibility of rural populations to heat.

Lindeboom et al. [36] noted the possibility of gradual acclimatization as communities adapt to living in warmer climates. Generally, regions within the tropical zone are better acclimatized and less sensitive to heat compared to those in the mid-latitude or temperate zones. This is apparent in higher temperature thresholds observed in tropical climates such as the Northern part of Ghana with a threshold above 30 °C [37]. Other tropical places in Nouna, Burkina Faso [35], and Bangladesh [15,34,36] had similarly high temperature thresholds (see Table 1). Temperature thresholds for locations in the mid-latitudes were lower. In the southern part of the US, classified as the warmest region in the country [53], temperatures above 28 °C increased mortality [16]. The thresholds were much lower for Hubei [40], Jiangsu [20], and Tibet [39] provinces of China. Even though rural residents in tropical countries seem better acclimatized to warm climates [35], their low adaptive capacity due to limited resources to cope with heat [15,36,37,39] increases their vulnerability. For instance, most houses in rural Matlab Bangladesh were described as “roofed and walled with corrugated iron sheets” [36], making residents more prone to heat effects. Informal settlements [37], common in rural areas of developing countries, can lead to overcrowding, thus impeding adequate ventilation and increasing pressure on limited rural health resources.

Some studies computed the heat vulnerability index by controlling for effect modifiers, such as average years of education, percentage of people ≥65 years old, number of air conditioning units per household, number of beds in health institutions per 1000 people [20], rurality, and deprivation [33]. Chen et al. [20] found a significant negative correlation between urbanicity and the heat vulnerability index in Jiangsu Province, China, while a study conducted in England and Wales found no correlation [33]. Previous studies have also found higher heat-related mortality risks in individuals with lower or no education [20,39,40], lower prevalence of air conditioning [1,20], as well as inadequate hospital infrastructure [20,40]. A majority of these individuals reside in rural areas.

There were inconsistencies in the definitions of rural areas across studies. Some studies just mentioned “rural” with no definition. Within the United States, for example, the Census Bureau’s definition of “rural” varies state-to-state. Further, the character of “rural” regions may depend greatly upon a country’s level of economic development, per the United Nations’ classification system [30]. To explore this point, a separate sensitivity analysis was conducted for developed and developing countries, as shown in Figure 5 and Figure 6. Heat-related mortality risk was higher in developing countries as compared to developed countries (i.e., 3.6% and 2.0%, respectively), consistent with the lack of infrastructure in developing countries.

We acknowledge additional limitations of this study. Our comprehensive search of international epidemiological studies on the association between temperature and mortality only located 14 studies, with the same temperature metric (daily mean) being used in eleven studies. The lack of meteorological data attributed to the paucity of weather stations in rural locations may have contributed to the limited number of studies we used for the meta-analyses. A further implication of widely spaced monitoring stations is that the exposure variable will not capture dynamic changes in temperature that occur in space, resulting in misclassification of exposure [16]. This misclassification is almost certain to be nondifferential (“random”) with respect to the outcome variable, mortality. Thus biased toward the null, relative risks derived from studies included in our meta-analysis may underestimate the true magnitude of heat’s effect on mortality.

With respect to the outcome variable, patterns of mortality in response to extreme heat are influenced by the underlying prevalence of temperature-sensitive diseases in a population. Epidemiologic studies of the impacts of extreme heat are most likely to find statistically significant increases in diseases that are already common. Although more common in urban areas, cardiovascular disease is a leading cause of death in rural areas world-wide [22]. Small increases above large, stable baseline rates—if caused by heat—are more likely to be detectable by statistical significance testing. None of the studies we reviewed differentiated among the various causes of death due to cardiovascular disease (i.e., stroke, MI, etc.), an area ripe for further investigation.

In addition, although respiratory mortality may be another potential outcome of interest, the current evidence concerning the association between heat and respiratory mortality is relatively weak. For instance, Hashizume et al. [15] found an increase in respiratory mortality only at low temperatures, unrelated to heat; and in their study encompassing a 16-year period, Azongo et al. [37] inferred that respiratory deaths in children were only tangentially related to heat, and more directly related to periods of high precipitation (along with diarrhea and malaria).

## 5. Conclusions

This study assessed heat-related mortality risks among rural populations worldwide through a comprehensive review of epidemiologic literature and meta-analysis. Fourteen epidemiological studies of the association between high temperature and mortality among rural populations were identified. These studies were conducted in eight countries on four continents between 2006 and 2017. Among the 14 studies, eleven using the daily mean metric for temperature were included in the random effects meta-analysis. The pooled estimates of relative risks (RRs) for all-cause and cardiovascular mortality were 1.030 (95% CI: 1.013, 1.048) and 1.111 (95% CI: 1.045, 1.181) per 1 °C increase in daily mean temperature, respectively. We found considerable heterogeneity in studies for cardiovascular mortality (I^2^ = 59.4%, *p* < 0.05), but not for all-cause mortality (I^2^ = 0%, *p* < 0.05). The combined risk of excess heat-related mortality in rural populations appears to be not smaller than those reported in urban populations, suggesting that being a rural resident does not make one less vulnerable to heat. We also found that the excess mortality risk was roughly 1.8 times higher among the developing nations than the developed nations included in the meta-analysis.

A key limitation of this study is the relatively small number of available studies focusing on rural populations worldwide. Lower population density and more dispersed weather stations are some of the factors that challenge quantitative studies of the relationship between temperature and mortality in rural areas [54]. Rural areas may also struggle with incomplete death registration, particularly in less developed regions [55]. However, further investigations of heat-related mortality in rural populations are certainly warranted. Future studies could also examine other causes of death, such as respiratory causes, in addition to premature mortality attributed to high temperature, all aimed at developing better public health interventions for heat risk management in rural areas.

## Figures and Tables

**Table 1:** Characteristics of selected studies that examined temperature effects on all-cause and cause-specific mortality.

Studies (Year Published)	Study Period	Location	Effect Estimate [RR per °C (95% CI)]	Potential Confounding Factors	Temperature Threshold (°C)	Mortality Outcome (s)	Study Population	Lag Period (Days)
***Studies using daily mean temperature***								
Hajat et al., (2006) [33]	1993–2003	England & Wales	1.020 (1.010, 1.030)	Ozone, PM_2.5_, seasonal varying factors, influenza epidemics	17–18	All-cause	N/A	0–1
Hashizume et al., (2009) [15]	1994–2002	Matlab, Bangladesh	1.629 (1.232, 2.152)	Seasonality	30	Cardiovascular	220,000	0–1
Burkart et al., (2011) [34]	2003–2007	Bangladesh	1.044 (0.990, 1.098)	Trend, season, day of the month and age	28.9	All-cause	~1,000,000	0–1
Diboulo et al., (2012) [35]	1999–2009	Nouna, Burkina Faso	1.026 (1.001, 1.052)	Time trends and seasonality	30	All-cause	90,000	0–1
Lindeboom et al., (2012) [36]	1983–2009	Matlab, Bangladesh	1.002 (1.001, 1.003)	Trend and seasonality	29	All-cause	225,002	0–1
Azongo et al., (2012) [37]	1995–2010	Northern Ghana	1.018 (1.007–1.029)	Time trends and seasonality	30.7	All-cause	N/A	0–1
Urban et al., (2014) [38]	1994–2009	Czech Republic	1.085 (1.05, 1.12)	Winter days during six epidemics	23.5	Cardiovascular	3,400,000	N/A
Bai et al., (2014) [39] Bai et al., (2014) *	2008–2012 2008–2012	Naidong (Tibet), China Jiangzi (Tibet), China	1.047 (0.181, 1.144) 1.063 (0.167, 2.020) 1.037 (0.222, 1.121) 1.134 (0.206, 2.217)	Seasonality and long-term trend Seasonality and long-term trend	15.3 11.8	All-cause and cardiovascular All-cause and cardiovascular	N/A N/A	0–1 0–1
Chen et al., (2016) [20]	2009–2013	Jiangsu Province, China	1.032 (1.028, 1.037)	Long-term trends and seasonality	24.1	All-cause	73,900,000	N/A
Lee et al., (2016) [16]	2007–2011	Georgia, North & South Carolina, U.S.	1.021 (0.995, 1.047)	PM_2.5_, age, race education, rural location	28.0	All-cause	N/A	N/A
Zhang et al., (2017) [40]	2009–2012	Hubei, China	1.14 (1.02, 1.26)	Long-term and seasonal trends	27.7	All-cause	6,700,000	0–2
***Studies using daily maximum temperature***								
Ingole et al., (2015) [41]	2003–2012	Vadu, India	1.36 (1.30, 1.42)	Day of the week, secular trends and other time-varying confounding factors	39.0	All-cause	131, 545	0
Madrigano et al., (2015) [42]	1988–1999	New York, New Jersey, Connecticut, U.S.	1.007 (1.006, 1.008)	Ozone	21.1	All-cause	N/A	N/A
***Studies using weekly mean temperature***								
Alam et al., (2012) [43]	1983–2009	Abhoynagar, Bangladesh	1.0 (no risk)	Rainfall	23.0	All-cause	34,774	0–3 weeks

* Same study in 2 locations.

**Table 2:** Effect size estimates of studies using daily mean temperature for all-cause mortality.

Study (Year)	Location (Country)	Effect Size (95% Confidence Interval)			Weight%
Hajat (2006)	England & Wales	1.020	1.010	1.030	14.20
Burkart (2011)	Bangladesh	1.044	0.990	1.098	6.43
Diboulo (2012)	Nouna, Burkina Faso	1.026	1.001	1.052	11.72
Lindeboom (2012)	Matlab, Bangladesh	1.002	1.001	1.003	14.86
Azongo (2012)	Northern Ghana	1.018	1.007	1.029	14.06
Bai (2014)	Naidong, China	1.047	0.20	1.144	2.75
Bai (2014) *	Jiangzi, China	1.037	0.222	1.121	3.52
Chen (2016)	Jiangsu Province, China	1.032	1.028	1.037	14.35
Lee (2016)	Southeast U.S.	1.021	0.995	1.047	13.77
Zhang (2017)	Hubei, China	1.140	1.020	1.260	4.35
**Pooled (I^2^ = 0.0%; *p* = 0.001)**		**1.030**	**1.013**	**1.048**	**100**

Note: Weights are from random effects analysis.

**Table 3:** Effect size estimates of studies using daily mean temperature for cardiovascular mortality.

Study (Year)	Location	Effect Size (95% Confidence Interval)			Weight%
Hashizume (2009)	Matlab, Bangladesh	1.629	1.232	2.152	5.10
Urban (2014)	Czech Republic	1.085	1.05	1.12	32.28
Bai (2014)	Naidong, China	1.063	0.20	2.02	23.80
Bai (2014) *	Jiangzi, China	1.134	0.206	2.217	38.82
**Pooled (I^2^ = 59.4%; *p* = 0.001)**		**1.111**	**1.045**	**1.181**	**100**

Note: Weights are from random effects analysis.

**Table 4:** Effect size estimates of studies in developing countries.

Study (Country)	Effect Size (95% Confidence Interval)			Weight%
Burkart (Bangladesh)	1.044	0.990	1.098	18.29
Diboulo (Burkina Faso)	1.026	1.001	1.052	4.79
Lindeboom (Bangladesh)	1.002	1.001	1.003	5.99
Azongo (Ghana)	1.018	1.007	1.029	10.08
Bai (China)	1.047	0.200	1.144	18.55
Bai * (China)	1.037	0.222	1.121	16.09
Chen (China)	1.032	1.028	1.037	18.99
Zhang (China)	1.140	1.020	1.260	7.23
**Pooled (I^2^ = 0.0%; *p* = 0.004)**	**1.036**	**1.012**	**1.061**	**100**

Note: Weights are from random effects analysis.

**Table 5:** Effect size estimates of studies in developed countries.

Study (Country)	Effect Size (95% Confidence Interval)			% Weight
Hajat (England & Wales)	1.020	1.010	1.030	87.1
Lee (USA)	1.021	0.995	1.047	12.9
**Pooled (I^2^ = 0.0%; *p* = 0.000)**	**1.02**	**1.011**	**1.03**	**100**

Note: Weights are from random effects analysis.
